# The Integrated Approach in Patients with Spinal Muscular Atrophy in the Era of Early Diagnosis, Etiopathogenic Therapies and Multidisciplinary Standards of Care and Rehabilitation Interventions Leads to New Phenotypes

**DOI:** 10.3390/life15111731

**Published:** 2025-11-10

**Authors:** Madalina Cristina Leanca, Andrada Mirea, Georgiana Nicolae, Andrei Capitanescu, Constantin Munteanu, Gelu Onose

**Affiliations:** 1 Faculty of General Medicine, University of Medicine and Pharmacy “Carol Davila”, 37 Dionisie Lupu Street, 020021 Bucharest, Romania; mada_mada332@yahoo.com (M.C.L.); andrada.mirea@gmail.com (A.M.); dr.georgiananicolae@gmail.com (G.N.); gelu.onose@umfcd.ro (G.O.); 2 National University Center for Children Neurorehabilitation “Dr. Nicolae Robanescu”, 44 Dumitru Minca Street, 041408 Bucharest, Romania; 3 Children’s Emergency Clinical Hospital “Maria Sklodowska Curie”, 20 Constantin Brancoveanu, 075534 Bucharest, Romania; andreicapitanescu@gmail.com; 4 Department of Biomedical Sciences, Faculty of Medical Bioengineering, University of Medicine and Pharmacy “Grigore T. Popa”, 700115 Iasi, Romania; 5 Teaching Emergency Hospital “Bagdasar-Arseni”, 12 Berceni Avenue, 041915 Bucharest, Romania

**Keywords:** spinal muscular atrophy, new phenotypes, disease modifying therapy, standards of care

## Abstract

Novel targeted therapies have transformed spinal muscular atrophy from a condition with a predictable, severe course into a more heterogeneous disorder with a range of new clinical phenotypes and outcomes. The emergence of new phenotypes in spinal muscular atrophy is a recent development in the field. The introduction of new etiopathogenic pharmacological treatments have significantly altered the natural history of the disease, leading to previously unseen clinical presentations and outcomes. Materials and Methods: We observed a cohort of 104 patients (children and adolescents), considering the number of SMN2 gene copies, the use of respiratory ventilation support devices and gastrointestinal support, and finally, their evolution on clinical-functional scales with physical therapy and rehabilitation interventions. With the increasing availability of effective therapies for spinal muscular atrophy, outcome measurement in clinical practice and research requires highly sensitive and reliable tools. In this study, motor function was systematically evaluated using two validated scales—the Children’s Hospital of Philadelphia Infant Test of Neuromuscular Disorders (CHOP INTEND) and the Hammersmith Functional Motor Scale Expanded (HFMSE)—which are specifically designed to capture incremental changes in motor skills across the spectrum of SMA severity and age groups. Results: The median scores on the validated tools steadily increased over the 24 months of follow-up. Starting from 29 at baseline, the scores rose to 36 at 6 months, then to 39 at 12 months, 43 at 18 months, and 44.5 at 24 months. The Friedman test showed that these changes were statistically significant (*p* < 0.01). Moreover, each follow-up score was significantly higher than both the baseline and the previous time point (all *p* < 0.01), showing continuous improvement over time. Conclusions: These findings reveal that the development of new SMA phenotypes is closely linked to the stage of disease at which treatment is initiated. Earlier intervention consistently enables patients to acquire previously unattainable motor skills. Consequently, enhancing diagnostic precision and expediting therapy initiation is crucial for maximizing clinical benefits and facilitating optimal functional outcomes.

## 1. Introduction

5q Spinal Muscular Atrophy (SMA) is a rare (orphan) hereditary autosomal recessive neurodegenerative disease [1]. The disease affects motor neurons in the anterior horn of the medullary, often those in the brainstem, and leads to their death with loss of muscle mass and motor deficit [2]. SMA is one of the most common paediatric recessive genetic diseases. In Europe, the incidence is 1 in 5000–10,000 live births and the carrier frequency is ~1 in 50—however the frequency is lower in some countries, especially in sub-Saharan Africa [3]. SMA is caused by deletion or mutation of the survival motor neuron 1 (SMN1) gene. The nearly identical SMN2 gene fails to generate adequate levels of functional SMN protein due to a splicing defect [4]. Several types of SMA have been described and they vary in severity, but type I SMA accounts for ~50–60% of incident SMA and is the most severe, usually resulting in death before 2 years of age [5]. The clinical spectrum of SMA also comprises less severe forms, including—types II, III, and IV—with each presenting distinct onset ages and motor abilities. Type II SMA typically manifests before 18 months of age, allowing affected children to sit independently but presenting challenges in standing. Type III usually begins after 18 months, enabling standing or ambulation, and type IV arises in adolescence or adulthood, with ambulatory patients and the mildest progression [6]. In contrast, Duchenne muscular dystrophy (DMD) is a severe pediatric neuromuscular disorder caused by dystrophin gene mutations that result in absent or defective dystrophin protein within muscle fibers [7]. Unlike SMA, DMD features progressive degeneration and necrosis of muscle tissue—despite intact motor neurons—and is characterized by muscle wasting, calf hypertrophy, and replacement of muscle with fat and connective tissue. DMD patients usually reach early motor milestones but later experience progressive weakness due to intrinsic muscle pathology [8]. A comparison between SMA and DMD highlights how advances in gene-targeted therapies and multidisciplinary care are transforming management for pediatric neuromuscular disorders. SMA now benefits from SMN-targeted gene therapies like onasemnogene abeparvovec and antisense oligonucleotides, while DMD treatment is being revolutionized by exon-skipping drugs and gene replacement strategies [9]. Both diseases have entered a new therapeutic era, where precision genetic medicine and coordinated multidisciplinary care optimize patient outcomes and fundamentally alter natural history.

Three treatments (Onasemnogene abeparvovec, nusinersen and risdiplam) that increase SMN expression by distinct molecular mechanisms, administration routes and tissue biodistributions have received regulatory approval with others in clinical development. Onasemnogene abeparvovec focuses on SMN1 gene modification and nusinersen and risdiplam focus on replacement or RNA modulation [10]. The advent of new therapies is redefining standards of care most patients in many countries are treated with one of the new therapies, leading to the identification of emerging new phenotypes of SMA and a renewed characterization of demographics owing to improved patient survival [4].

These new therapeutic approaches and the standardization of care have changed the natural history of the disease, increasing survival in severe forms, and showing the progression of all types of SMA [11]. New phenotypes of SMA now cross over traditional subtypes of SMA, so it is more appropriate to rely on a combination of age of onset, number of SMN2 copies, and age at initiation of drug treatment, rather than traditional subtypes, to define a clinical phenotype of SMA [12].

Treatment decisions should be based on individualized assessment, performed by a specialist experienced in treating patients with AMS, weighing the benefits of treatment for the patients, in relation to its potential risks [6]. The initial clinical evaluation should be performed when the patient is in stable condition, without intercurrent illness, in order to correctly reflect their motor and respiratory function [13].

The introduction of novel targeted therapies has significantly altered the natural course of SMA, resulting in new phenotypic presentations [14]. Over time, clinicians will increasingly face two populations of SMA: pre-symptomatic newborn treated within the first few weeks of life, and symptomatic older infants, children, and adult [15]. Each group of patients will respond differently to these drugs and expectations will need to be aligned accordingly [16]. Counseling patients and children will be more complex as additional drugs for treatment of SMA are approved by regulatory authorities [17].

Nusinersen is an antisense oligonucleotide (ASO) [18] that increases the proportion of exon 7 inclusion in the transcription process of the messenger ribonucleic acid (mRNA) encoding the survival motor neuron 2 (SMN2) protein. It does this by binding to an intronic splice silencing site (ISS-N1) found in intron 7 of the pre-messenger ribonucleic acid (pre-mRNA) of SMN2 [19]. Through binding, ASOs displace assembly factors that normally suppress gene expression, leading to the retention of exon 7 in SMN2 mRNA and translation into full-length functional SMN protein when SMN2 mRNA is produced [20]. Administration is intrathecal, starting with a loading dose of four injections as follows: one intrathecal injection on day 0, day 14, day 28, and day 60, followed by a maintenance dose of one intrathecal injection every 120 days [19]. Each administration of nusinersen, whether a loading or maintenance dose, consists of a single 12 mg dose [21].

Risdiplam is an orally administered small-molecule splicing modifier that increases the inclusion of exon 7 in SMN2 pre-mRNA, leading to increased production of full-length, functional SMN protein systemically [22]. Risdiplam binds to SMN2 pre-mRNA and enhances the recruitment and binding affinity of the U1 small nuclear ribonucleoprotein (U1 snRNP) to the weakened 5′ splice site at exon 7. This compensates for a splicing defect that normally causes exon 7 skipping in SMN2 transcripts [23]. By promoting exon 7 inclusion, risdiplam increases the proportion of full-length SMN2 mRNA transcripts, which are translated into functional SMN protein, compensating for the loss of SMN1 protein in SMA [24]. Its oral bioavailability and ability to cross the blood-brain barrier allow risdiplam to exert systemic effects, increasing SMN protein production in the central nervous system and in peripheral tissues like muscles and organs [25].

Onasemnogene abeparvovec is a gene therapy designed to introduce a functional copy of the survival of motor neuron gene (SMN1) into transduced cells to address the primary monogenic cause of the disease. By providing an alternative source for SMN protein expression in motor neurons, it is expected to promote the survival and function of transduced motor neurons. Onasemnogen abeparvovec is a recombinant, replication-incompetent adeno-associated virus (AAV) vector that utilizes the AAV9 virus capsid to deliver a stable, fully functional human SMN transgene. The AAV9 capsid has been shown to cross the blood-brain barrier and transduce motor neurons [26]. The SMN1 gene present in onasemnogene abeparvovec is designed to remain fixed as episomal DNA in the nucleus of transduced cells and is expected to be stably expressed over a long period of time in postmitotic cells [27]. The AAV9 virus is not known to cause disease in humans [28]. AAVs, including AAV9, are considered non-pathogenic [29]. The transgene is introduced into target cells as a self-complementary double-stranded molecule [30]. Expression of the transgene is driven by a constitutive promoter (composed of the cytomegalovirus enhancer and the chicken β-actin hybrid promoter), which drives continuous and sustained expression of the SMN protein [31]. Evidence of the mechanism of action is supported by non-clinical studies and biodistribution data in humans [32]. Gene therapy administration is by intravenous infusion, a single dose in life [33].

The emergence of these new phenotypes presents challenges in classification and management. These challenges include an undefined spectrum of severity-the phenotype of SMN1-associated SMA now spans a continuum without clear delineation of subtypes. Additionally the long-term effects of new targeted treatments are unknown, potentially leading to evolving phenotypes over time. Finally disparities in access to novel therapies may result in varied phenotypes across different populations [34].

As research progresses and more patients receive targeted therapies, our understanding of SMA phenotypes will continue to evolve, necessitating ongoing updates to classification systems and management strategies [35].

This study aims to describe longitudinal clinical outcomes among Romanian children with spinal muscular atrophy who have received early and sustained disease-modifying therapies in order to investigate the impact of SMN2 copy number, timing and type of therapeutic intervention and comprehensive supportive care on both motor and respiratory functions, as well as to analyze the development and transformation of SMA phenotypes within the context of widespread newborn screening and the adoption of innovative treatment strategies.

## 2. Materials and Methods

Within the scope of our work, we present a cohort of patients characterized by stratification according to SMA type and disease stage, utilizing the validated motor assessment tools, The Children’s Hospital of Philadelphia Infant Test of Neuromuscular Disorders (CHOP INTEND) and Hammersmith Functional Motor Scale Extended (HFMSE), in alignment with established standards at Romanian clinical hospitals. In our research, it is important to consider the advantages and limitations of these assessment scales regarding sensitivity, specificity, clinical utility, and suitability for different patient groups. Additionally, we highlight the distinctive national achievements of the National University Center for Children Neurorehabilitation “Dr. Nicolae Robanescu” listed below:

- The first center in Romania to administer, in October 2018, the disease-modifying therapy Nusinersen Spinraza reimbursed by the National Health Program—SMA;
- The first center in Romania to administer Risdiplam treatment in April 2020 within the Compassionate Use Program; we currently have 45 patients in treatment reimbursed through the National Health Program—SMA;
- The first center in Romania to administer gene therapy with Onasemnogene abeparvovec in July 2020 under the Compassionate Use Program; currently, four patients have received gene therapy through the National Health Program—SMA;
- The center with the most pediatric patients in the country, with 135 patients registered for etiopathogenic treatment and standards of care;
- The only center in Romania that is carrying out the neonatal screening pilot study for SMA from August 2022 with 28 maternity hospitals in Bucharest and neighboring counties.

The infrastructure of the genetics laboratory of the National University Center for Children Neurorehabilitation “Dr. Nicolae Robanescu” was previously dedicated to the molecular diagnosis of COVID-19, and the specialists in the laboratory chose the working technique. The multidisciplinary medical team—12 medical specialties alongside nurses, physiotherapists, psychologists, speech therapists, social workers—had at their disposal the material base of the Center to take over and treat patients with SMA detected before symptoms through screening: this included an outpatient consultation office, day and continuous hospitalization, imaging department and functional explorations, treatment base that includes robotic devices for hydro kinetotherapy. Over a period of three years, 60,000 newborns were screened, constituting almost 40% of the country’s newborn population. The project is ongoing and will continue until the success of its national implementation, estimated to be in 2025. The feasibility study allowed the identification of 13 children out of the 60,000 tested in the presymptomatic stage. Our efforts were recorded, tracked and encouraged by European organizations dedicated to neonatal screening for SMA.

### 2.1. Study Design and Objective

We performed a retrospective observational study in a pediatric population with SMA in Romania. The main characteristics of our research center are the multidisciplinary team (doctors, nurses, physiotherapists) applying the standard of care in SMA, all the equipment for the management and treatment of these children and the infrastructure for rapid diagnosis (newborn screening study pilot). The primary aim of this study is centered on the profound disability caused by SMA, which, in severe cases, may lead to death. This investigation also considers the marked improvements in patient outcomes observed in recent years following access to etiopathogenic treatments. Specifically, the objectives are to stratify patients by SMA type, disease stage, and SMN2 copy number; to monitor clinical and motor function using validated assessment scales; to evaluate dependence on ventilatory and nutritional support; and to analyze the relationships among these factors in order to better inform prognosis and treatment strategies.

### 2.2. Participants and Recruitment

This study was conducted over 3 years between 1 July 2022 and 30 June 2025. A total of 3 groups of patients were followed: group 1 included patients with type I SMA (51 patients), group 2 included patients with type II SMA (36 patients), and group 3 included patients with type III SMA (17 patients).This study relied on existing data and records, which can impact the recruitment process and introduce potential biases. Ensuring the completeness and accuracy of existing records is crucial for reliable participant selection.

Inclusion Criteria

✓ Genetically confirmed diagnosis of SMA with-SMN1 gene homozygous deletion;
✓ Specific SMA types (type I, II or III);
✓ Ability to perform certain functional assessments, depending on the study’s objectives;
✓ Treatment initiated in our clinic (Nusinersen or Risdiplam or Onasemnogene Abepravovec);
✓ Signing of informed consent by the patient or legal representative.

Exclusion criteria

✓ Patients without genetic confirmation of the disease, including SMA non5q;
✓ Refusal to sign the informed consent by the patient/legal representative.

### 2.3. Data Collection

#### 2.3.1. Sociodemographic and Physical Condition

All 3 groups were followed up according to the age at which the diagnosis was made and etiopathogenic treatment was initiated (nusinersen, risdiplam or onasemnogene abeparvovec), the number of SMN2 copies and the statistical correlation with the disease phenotype after treatment. Besides these treatments, which should be administered as soon as symptoms occur, it is critical to follow correct and comprehensive standards of care, one of which is physical therapy [36]. SMA has multisystemic effects beyond muscle weakness: cardiovascular, metabolic, gastrointestinal and other organs (kidney, liver). Also, family history is important because nearly all cases (95%) result from inherited mutations, though spontaneous mutations rarely occur [37]. These aspects are not included in this research and these items contain answers for future studies.

#### 2.3.2. Tools for Quantified Assessment of Motor Function

Patients were assessed by licensed physical therapists with pediatric SMA experience from the National University Center for Children Neurorehabilitation “Dr. Nicolae Robanescu” using CHOP INTEND [38](for type I) and HFMSE [39] for types II and III. The choice of scale is critical and should be driven by the clinical objective (monitoring natural history, evaluating response to therapy, or stratifying for trials) and the patient’s functional status, age, and SMA type. No single scale is optimal for all scenarios. Multipronged assessment improves overall disease monitoring and because each SMA clinical-functional scale possesses unique sensitivity, specificity, and usability characteristics, clinicians and researchers must select and interpret outcome measures thoughtfully, considering disease stage, therapeutic goals, and patient phenotype. The mosaic of unique performance features across scales means that a comprehensive approach, using multiple scales, or innovating new scales combining the strengths of existing ones, yields the most accurate and clinically meaningful insights for both individual care and clinical research in SMA.

The CHOP INTEND is a well-established assessment tool for evaluating motor function in infants with SMA, particularly those diagnosed with Type 1 [40]. Comprising 16 items, it assesses various motor activities—including spontaneous movements of the arms and legs, hand grip, head control, hip adduction, shoulder and elbow flexion, and antigravity actions. Each item captures both reflexive and voluntary movements, focusing on strength and motor control in infants with significant muscle weakness, as commonly seen in SMA Type 1. Scoring is based on a scale from 0 (no response) to 4 (full response) for each item, yielding a total possible score between 0 and 64 [41]. The reliability of the CHOP INTEND has been demonstrated across populations of children with SMA-I, other neuromuscular disorders, and typically developing infants. As such, it serves as an effective measure of motor skills for both clinical monitoring and research purposes in this population [38,42].

The HFMSE is a widely used assessment tool for evaluating motor function in individuals with SMA, particularly types II and III [43]. This scale is an extension of the original Hammersmith Functional Motor Scale (HFMS), which was designed to assess non-ambulant SMA patients. The HFMSE evaluates gross motor function in both ambulant and non-ambulant SMA patients, using 33 tasks such as rolling, sitting, crawling, standing, and walking. Each task is scored 0–2, with a total possible score between 0 and 66. The HFMSE assesses gross motor function in people with SMA using 33 tasks like rolling, sitting, crawling, standing, and walking. Each activity is rated on a 3-point scale, and total scores can range from 0 to 66 [38]. When scoring the HFMSE, it is important to note that musculoskeletal contractures can make some assessments difficult, particularly those at the Achilles tendon, hamstrings, and hip flexors. By carefully considering the impact of contractures, assessors can ensure more accurate and consistent HFMSE scoring, providing a clearer picture of a patient’s functional abilities and disease progression in SMA [44].

Both scales provide a detailed, objective measurement of motor ability, with higher scores reflecting better function and lower levels of disability [45]. They are sensitive to changes over time and detect improvements or decline in motor function, making them ideal for tracking disease progression or response to therapy [46]. Although some items related to respiratory and bulbar function present challenges, the scale effectively complements traditional motor assessments by capturing the dimension of functional independence [47].

The characteristics in SMA studies include SMA type, age of onset, motor function, genetic factors (SMN1 deletions, SMN2 copy numbers, hybrids), disease progression markers (respiratory function, swallowing function) and treatment responses [48,49].

#### 2.3.3. Accessibility of Disease-Modifying SMA Therapy in Romania

**Figure 1:**
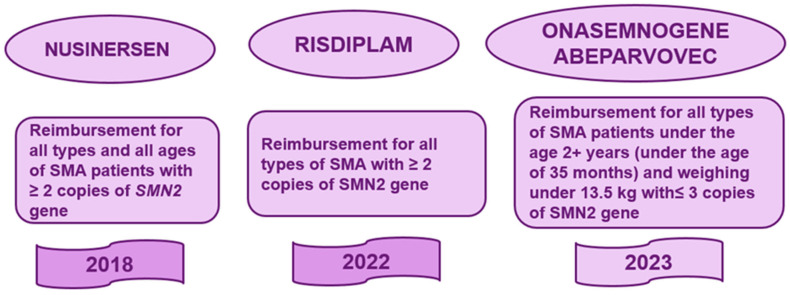
Disease-modifying therapies available in Romania, with approval years indicated in the final row. Approvals are governed by Government Decision 720/2008 (with subsequent amendments) and Minister of Health Orders 499/564/2021 (with their respective updates), setting the regulatory framework for spinal muscular atrophy treatment accessibility.

Currently, three therapies aimed at increasing SMN protein levels are available in Romania: onasemnogene abeparvovec, nusinersen and risdiplam. Regarding accessibility under the National Health Program for Rare Diseases for disease-modifying treatment, according to Government Decision 720/2008 and Ministry of Health Order 499/564/2021, with subsequent amendments and completions, patients can be divided into three treatment eras: (1) the nusinersen era between 2018 and 2022; (2) the era of transition, when treatment shifted from intrathecal nusinersen to oral risdiplam between 2022 and 2024; and (3) the era of gene therapy with onasemnogene abeparvovec, beginning in 2023 (Figure 1).

#### 2.3.4. Statistical Method

Statistical analysis was performed using SPSS “Statistical Package for Social Sciences” version 22.0 and Microsoft Excel 2010. Data analysis was conducted using non-parametric statistical methods appropriate for the study design and data distribution. The Friedman test was employed to analyze related samples across multiple conditions, given its suitability for repeated measures data. For comparisons involving two independent groups, the Kruskal-Wallis test was utilized to assess differences in distributions. Nominal variables are summarized as counts, while continuous variables are expressed as medians accompanied by interquartile ranges to reflect data dispersion. The null hypothesis of the Friedman test states that the median ranks are equal across the related groups, and if the test is significant, it indicates that there is a difference in at least one group compared to the others [50]. Friedman’s test statistic is often denoted as Q and its calculation involves summing ranks within each condition across subjects [51]. Assumptions of the tests were verified prior to analysis, and post hoc analyses were conducted when applicable to further explore significant findings [52].

## 3. Results

### 3.1. Cohort Characteristic

The cohort included 104 patients (50% male) with a median age of onset of 48 months predominantly with two or three SMN2 copies all treated with disease-modifying therapies. The characteristics of the cohort are presented in Table 1 and include distribution by sex and genetic diagnosis, namely the number of SMN2 copies, respiratory and nutritional status, all patients having homozygous deletions of SMN1 exon 7/8. The age of onset refers to the median age at which symptoms of spinal muscular atrophy (SMA) began in the studied cohort, with an interquartile range (IQR) from 9 to 106 months. This means half of the cohort developed symptoms between 9 and 106 months old, and the median was 48 months.

### 3.2. Motor Function

Motor function, evaluated using the CHOP INTEND scale for SMA Type I and the HFMSE for Types II and III, demonstrated progressive and statistically significant improvement following initiation of disease-modifying therapy. For CHOP INTEND, the median scores improved from 23 (IQR: 16–32) at baseline to 40 (IQR: 28–47.8) at 6 months, 42 (IQR: 35.2–52) at 12 months, 49 (IQR: 38.5–56) at 18 months, and 53 (IQR: 40.7–56.8) at 24 months. Similarly, HFMSE median scores increased from 32 (IQR: 23.7–45.7) at baseline to 33 (IQR: 28–47.3) at 6 months, 35 (IQR: 28–48.7) at 12 months, 35 (IQR: 29–49.3) at 18 months, and 36 (IQR: 29–49.3) at 24 months. All sequential assessments showed statistically significant improvement (*p* < 0.01). Collectively, these data reveal that overall median motor scores rose from 29 at baseline to 44.5 at 24 months, underscoring the sustained motor gains achieved over the course of therapy. In Table 2, the longitudinal changes in motor assessment scores (CHOP INTEND and HFMSE) in the treated SMA cohort are summarized. We are referring to changes and trends (improvements or declines) in motor function, as tracked by clinical assessments (CHOP INTEND and HFMSE), across multiple follow-up periods in a group of treated SMA patients. This analysis helps show how treatment affects motor abilities over time.

### 3.3. Influence of SMN2 Copy Number

**Figure 2:**
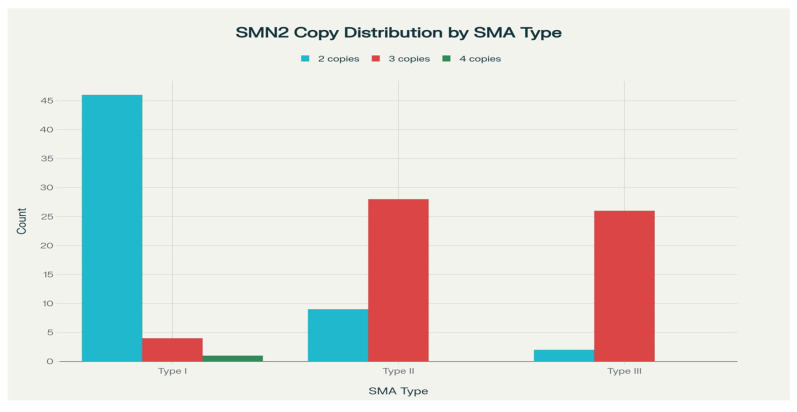
Distribution between clinical type of SMA and number of SMN2 copies.

The number of SMN2 copies is inversely correlated with the severity of the disease: higher SMN2 copy numbers generally correlate with milder phenotypes and the correlation in the studied group with all treated patients is represented in the diagram, so that there is a correlation between phenotype and the number of SMN 2 copies (see Figure 2). Lower SMN2 copy number (typically two) correlates with more severe SMA (type I: earlier onset, severe weakness, rapid progression). Higher SMN2 copy number (three or more) usually corresponds to milder phenotypes (type II or III: later onset, less severe weakness, and slower progression). 

**Figure 3:**
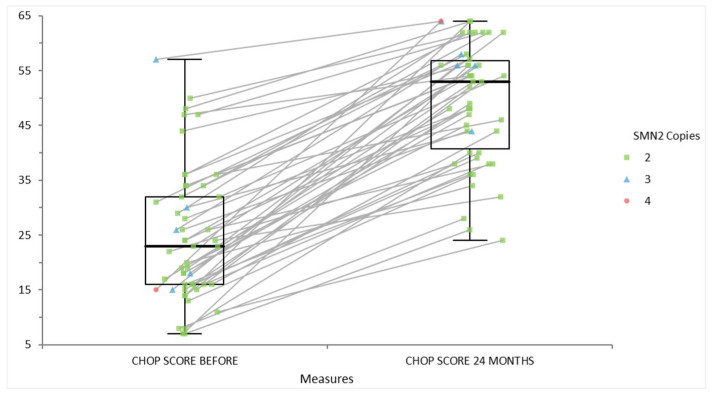
The functional score CHOP INTEND scale for individuals with SMA, stratified by their SMN2 copy number before and after 24 months following a therapeutic intervention.

Figure 3 depicts the individual changes in CHOP INTEND scores—an assessment of motor function—among patients with spinal muscular atrophy, grouped by SMN2 copy number, over a 24-month period. Each line connects a patient’s baseline score to their 24-month follow-up, while marker shapes and colors represent SMN2 copy numbers (green squares for two copies, blue triangles for three copies, and red dots for four copies). The boxplots summarize score distributions for all patients at each timepoint. At baseline, most patients, especially those with only two SMN2 copies, started with lower scores, indicative of more severe motor impairment. After 24 months of disease-modifying therapy, all groups showed a pronounced increase in CHOP INTEND scores, with the most substantial gains observed in those with initially more severe disabilities. This demonstrates both the therapeutic benefit of modern SMA treatments and the correlation between SMN2 copy number and motor function improvement, with higher copy numbers generally associated with better outcomes.

**Figure 4:**
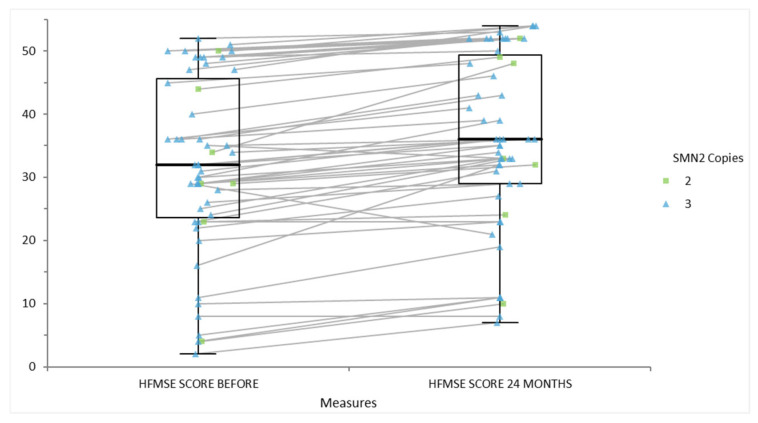
The functional scores on HFMSE scale for individuals with SMA, stratified by their SMN2 copy number after 24 months following a therapeutic intervention.

Figure 4 illustrates HFMSE scores before and 24 months after therapeutic intervention in individuals with spinal muscular atrophy (SMA), stratified according to SMN2 copy number. Each solid line traces the trajectory of functional score for an individual, connecting their baseline and follow-up values. The boxplots display the distribution of scores at each timepoint, with median, interquartile range, and extremities clearly shown. Points are color-coded to indicate SMN2 copy number: green squares represent individuals with two SMN2 copies, while blue triangles indicate those with three copies. The majority of patients, regardless of SMN2 copy number, exhibit stability or improvement in HFMSE scores over 24 months. Notably, individuals with three copies of SMN2 tend to occupy the higher end of the functional spectrum, suggesting a relationship between increased SMN2 copy number and better motor outcomes after intervention.

### 3.4. Respiratory and Nutritional Support

**Figure 5:**
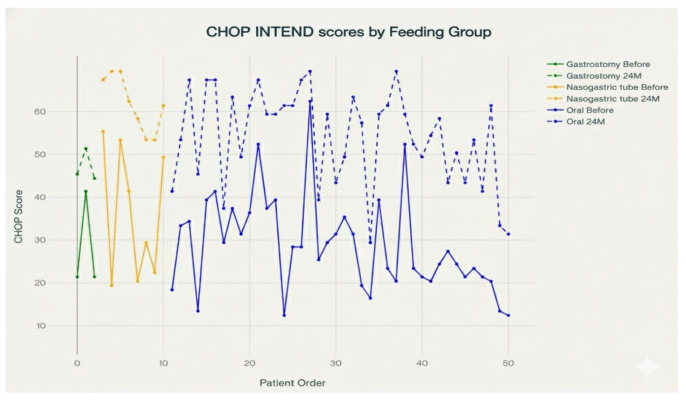
CHOP INTEND scores before and at 24 months by feeding method (oral, nasogastric tube, gastrostomy) for all patients.

Swallowing abnormalities in SMA are multifactorial, requiring tailored interventions to mitigate risks like aspiration pneumonia and malnutrition. Based on the provided data in our cohort, showing that 93 out of 104 SMA patients (89.4%) maintain oral alimentation, oral feeding remains the predominant nutritional approach in SMA, though it presents significant challenges that vary by disease severity and type. Nasogastric tubes are used in 7.7% (22/104) of SMA patients, while gastrostomy tubes are used in only 2.9% (3/104), indicating that most SMA patients (over 90%) manage without permanent feeding tube interventions. Figure 5 presents CHOP INTEND scores for individual patients at baseline and after 24 months, classified by feeding group: oral, nasogastric tube and gastrostomy. Distinct lines represent each group both before and after 24 months. The majority of patients in the oral feeding group demonstrate marked improvement or stability in their CHOP INTEND scores over time, visible in the upward shift from baseline (solid blue) to 24 months (dashed blue). For patients with nasogastric tube feeding, initial baseline scores are generally higher, and scores appear sustained over 24 months (dashed orange) with only minor fluctuations. In contrast, patients who required gastrostomy feeding tend to start with lower CHOP INTEND scores, and while some stabilization is seen at 24 months (dashed green), these scores remain lower compared to orally or nasogastrically fed groups. Overall, the graph indicates that feeding modality is associated with differences in baseline motor function and longer-term outcomes, as reflected in CHOP INTEND scores.

**Figure 6:**
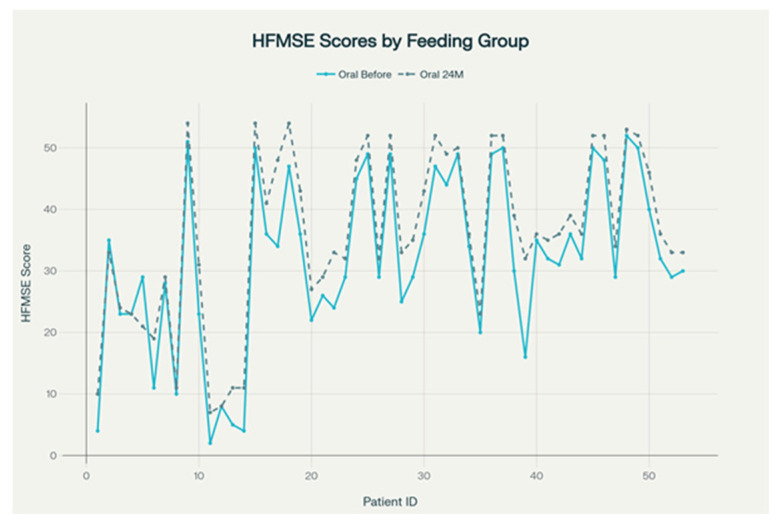
HFMSE score evolution over 24 months in SMA patients only oral feeding.

Figure 6 reveals individual patient trajectories for HFMSE scores at baseline and after 24 months, grouped by route of nutrition. In this cohort, all patients received oral feeding; no patients required nasogastric tube or gastrostomy. The plotted lines reveal a general trend of stability or improvement in HFMSE scores over the 24-month period, with most patients showing either maintained or increased functional motor abilities. These results suggest that, among orally fed patients, functional motor outcomes as measured by HFMSE tend to remain stable or improve over two years.

Non-invasive ventilation (NIV) and tracheostomy are both respiratory support options used in managing SMA, particularly due to respiratory muscle weakness leading to respiratory failure. Within the cohort, non-invasive ventilation (NIV) was required for 37 patients, corresponding to 35.6% of the total group. The majority, 67 patients (64.4%), did not require NIV. The relative frequency for patients requiring NIV was 0.356, while it was 0.644 for those not requiring NIV. The cumulative relative frequency reached 1.000, indicating that all patients were accounted for across both subgroups. This distribution highlights that most patients did not require non-invasive ventilation during the study period.

In the analyzed cohort, tracheostomy was required in only three patients, representing 2.9% of the total group. The majority, 101 patients (97.1%), did not require tracheostomy. The relative frequency for patients without tracheostomy was 0.971, while for those with tracheostomy it was 0.029. The cumulative relative frequency confirmed complete categorization of the cohort, highlighting that tracheostomy was a rare intervention among these patients.

The statistical analysis was performed (see Table 3). The data demonstrate a clear association between motor function and the need for non-invasive ventilation (NIV) in SMA patients, which closely aligns with published evidence. At baseline, those requiring NIV had much lower median motor scores (16 [10; 28]) than those not requiring NIV (32 [24; 44]), and this difference was statistically significant (*p* < 0.01). After 24 months of disease-modifying therapy, both groups improved and the difference in scores was no longer significant (44 [25; 53] vs. 46 [34; 54], *p* = 0.25).

**Figure 7:**
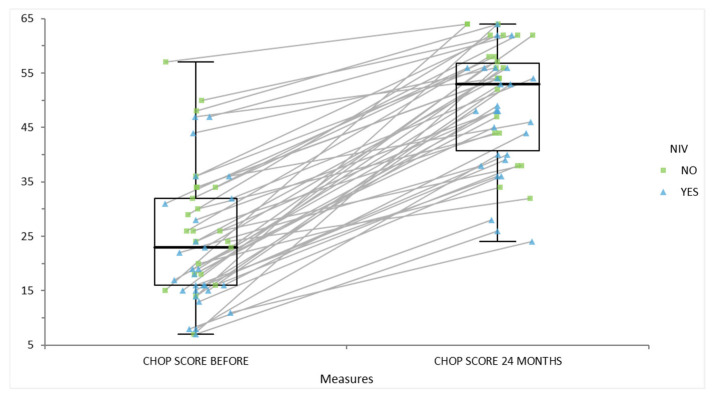
Score of motor scales CHOP INTEND before and after 24 months of disease-modifying therapy of patients with or without non-invasive ventilation.

Figure 7 illustrates individual changes in motor function, measured by CHOP INTEND scores, for patients with spinal muscular atrophy (SMA) over a 24-month period, stratified by their need for non-invasive ventilation (NIV). Each data point represents a patient’s score before treatment and at 24 months, with lines connecting paired measurements to highlight progression. Patients requiring NIV (indicated by blue triangles) generally started with lower CHOP scores compared to those not requiring NIV (green squares). Regardless of initial ventilation status, nearly all patients demonstrated substantial improvement in motor function by 24 months, as shown by the upward shift in scores and higher median boxplot values. This underscores the effectiveness of disease-modifying therapies in improving motor outcomes across varying severities of SMA and suggests that the gap in motor function between patients with and without NIV narrows significantly following treatment.

**Figure 8:**
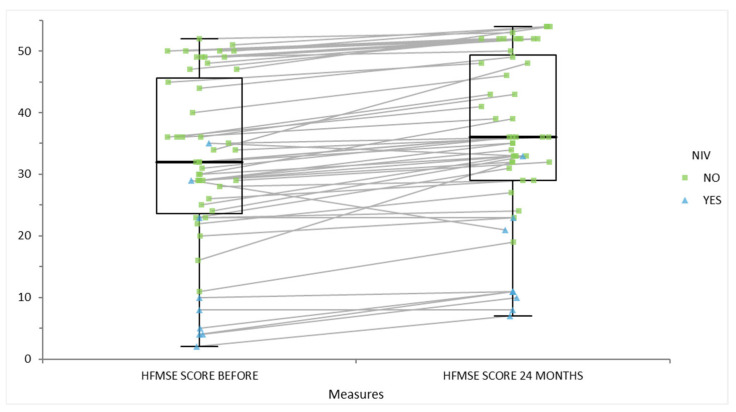
Score of motor scales HFMSE before and after disease-modifying therapy of patients with or without non-invasive ventilation.

Figure 8 displays changes in HFMSE scores for patients with spinal muscular atrophy over a 24-month period, differentiating individuals who required non-invasive ventilation (NIV; blue triangles) from those who did not (green squares). Each line connects a patient’s score before treatment (left) to their score after 24 months of therapy (right). The boxplots summarize the distribution for each timepoint. At baseline, patients requiring NIV generally had lower HFMSE scores, indicative of more severe motor impairment. After 24 months of disease-modifying treatment, most patients, regardless of NIV status, experienced an increase or stabilization in their scores, with a pronounced upward shift in both individual and group medians. The improvement is more variable among those requiring NIV; however, the overall narrowing of group differences highlights how therapy benefits patients across the clinical spectrum.

### 3.5. Phenotypic Evolution

**Figure 9:**
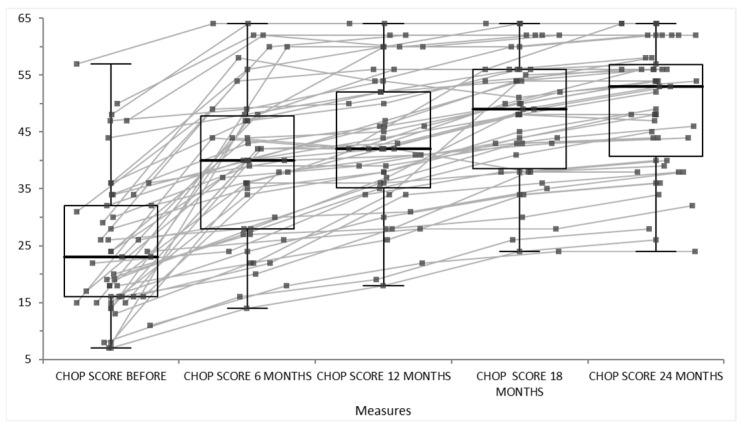
Score of CHOP INTEND scale before, at 6 months, at 12 months, at 18 months and 24 months of disease-modifying therapy.

**Figure 10:**
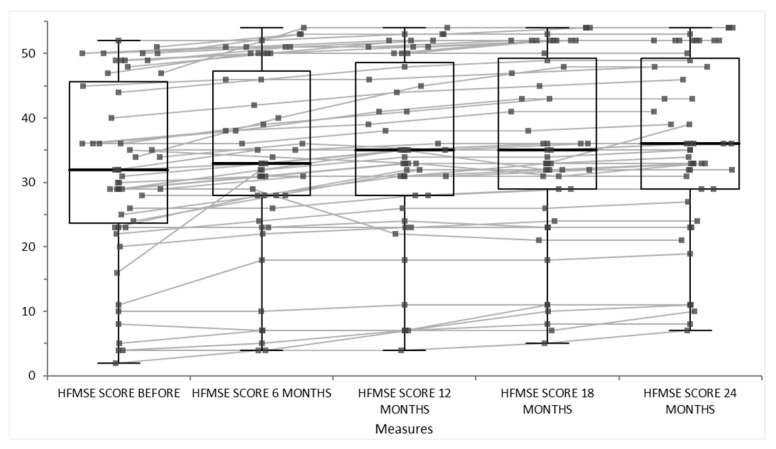
Score of HFMSE scale before, at 6 months, at 12 months, at 18 months and 24 months of disease-modifying therapy.

The patients treated with disease-modifying therapies such as Nusinersen, Risdi-plam or Onasemnogene Abeparvovec experience stabilization or modest improvement in motor function over 24 months, as measured by standard scales like CHOP INTEND, HFMSE. The results of our cohort are represented in Figure 9 for CHOP INTEND scale and Figure 10 for HFMSE scale. The CHOP INTEND scores significantly increased from baseline at every follow-up time point (6, 12, 18, 24 months), as evidenced by *p*-values less than 0.01. The increase over time suggests a consistent improvement in motor function or clinical status measured by CHOP INTEND. The median functional motor score increased consistently and significantly at every 6-month interval over 2 years of therapy, indicating progressive motor function gain in the first 6 months with higher motor scale scores. After that, the score of motor scales were still increased, not just stabilized, at 12 months and 18 months of disease-modifying therapy. The improvement from baseline is statistically significant (*p* < 0.01) at all follow-ups, and importantly, each stepwise increase vs the previous timepoint is also statistically significant. This suggests continuous incremental motor improvements over time. The statistical report and graphical summary interpretation of the HFMSE score data of our cohort data provided over 24 months showed a baseline median HFMSE score of 32 (with an interquartile range [IQR] roughly between 23.7 and 45.7). Over 24 months, scores gradually increased to a median of 36 [29; 49.3]. All follow-up assessments showed statistically significant improvement compared to baseline (*p* < 0.01).

Over 24 months of disease-modifying therapy in SMA patients, sustained, significant motor function improvements are shown, as measured by standardized functional motor scales (Figure 9, showing score of CHOP INTEND scale before, at 6 months, at 12 months, at 18 months and 24 months of disease-modifying therapy and Figure 10, showing score of HFMSE scale at 18 months and 24 months of disease-modifying therapy). Improvements are incremental and continuous, not just early gains that plateau in relation to the natural history of the disease. This pattern reflects an altered disease trajectory and suggests the long-term benefits of early and ongoing therapy.

**Figure 11:**
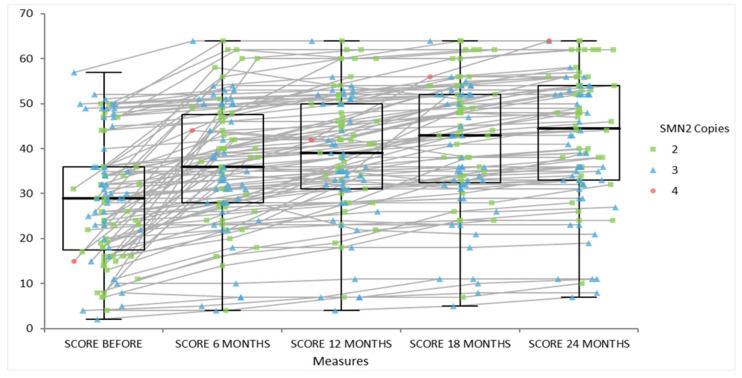
Progression of motor scale scores in SMA cohort by SMN2 copy number: baseline, 6 months, 12 months, 18 months and 24 months of follow-up.

After 24 months of disease-modifying therapy in SMA, we have shifted the trajectory: all patients (with two or three or four SMN2 copies) maintain or achieve higher motor function compared with historical (untreated) data. All patients and their scores at baseline, 6 months, 12 months, 18 months and 24 months are included in Figure 11 and the functional scores improved steadily from a median of 29 at baseline to 44.5 at 24 months. Friedman analysis was used to test all scores. Longitudinal observational data extending beyond 24 months report clinically relevant improvements or stabilization of motor abilities, particularly when treatment is initiated early. Patients with a range of SMN2 copy numbers have demonstrated either maintenance of baseline function or gains in motor milestones.

## 4. Discussion

These retrospective observational studies in SMA have been important in characterizing the disease’s natural history and assessing the long-term outcomes of various treatments, confirming sustained treatment efficacy. We measured changes in motor function, pulmonary capacity and nutritional status at multiple time points (baseline, 6 months, 12 months, 18 months, 2 years) after the innovative treatments. This comprehensive longitudinal research reveals whether new therapies alter natural history and highlight which outcomes are most responsive to interventions. Also, we explored potential new phenotypes linked to these innovative therapies. The results reflect significant and sustained motor function gains beyond the expected natural decline seen historically in untreated SMA populations. Early diagnosis, often enabled by newborn screening programs, maximizes opportunities for timely intervention that can significantly alter the disease course. Early initiation of treatment before irreversible motor neuron loss is critical for optimizing outcomes [53]. Greater benefits were seen in younger patients and those with higher baseline function. This improvement plays a critical role in enhancing patients’ quality of life and delaying or reducing respiratory complications. Functional motor score improvements serve as robust, clinically meaningful indicators of positive long-term outcomes in SMA, reflecting a significant shift from the previously relentless disease progression to a more modifiable course under modern therapies.

Participants evaluated using the CHOP-INTEND scale showed statistically significant improvements at every 6-month interval over 2 years. Improvements are progressive and sustained; the score increases stepwise rather than plateauing. This pattern suggests a positive and ongoing effect of whatever intervention or natural progression is being measured. The score trends are consistent with findings in recent SMA studies employing validated scales such as the HFMSE and CHOP INTEND [5,41]. These tools reliably capture subtle and sustained motor function improvements, which is particularly important in clinical trials and long-term monitoring of treated patients. This supports the conclusion that patients are achieving meaningful, incremental functional gains over 24 months, likely reflecting the positive impacts of modern therapeutic interventions combined with standardized assessment tools designed for sensitivity, range, and clinical relevance.

Patients receiving timely, disease-modifying therapies often experience a slowdown or halt in disease progression. In many cases, there is even measurable improvement in motor function, such as gaining milestones like sitting, standing, or walking, which were previously unattainable. With early pharmacological intervention combined with continuous multidisciplinary care, patients may reach a plateau where the disease course is stabilized. This stabilization is characterized by the long-term maintenance of existing motor skills and prevention of further functional decline.

The data strongly suggests that the intervention (likely an SMA-specific therapy) leads to significant functional improvements across all SMN2 copy numbers. While all groups improved, the relative improvement and the absolute final scores differed. Individuals with four SMN2 copies appear to achieve complete or near-complete functional recovery (reaching the presumed ceiling of 64) more consistently than those with two or three copies, though these groups also show very substantial gains. SMN2 copy number is a prognostic factor: the final scores at 24 months still show the trend that four-copy individuals achieve the highest (and perfectly uniform) scores, followed by two-copy, then three-copy. These findings reinforce the critical role of SMN2 copy number as a prognostic marker in SMA severity and as a predictor of treatment response, emphasizing the importance of individualized management and long-term monitoring.

Unlike DMD, where muscle fiber degeneration predominates, SMA patients in this cohort demonstrated improvement in motor scores following SMN-enhancer therapy, indicating a disease-modifying effect not typically observed in DMD. Our data reveal robust, sustained motor improvements across treated SMA populations—markedly distinct from the stagnating or regressive motor trajectory observed in DMD despite advances in supportive care. The capacity for phenotype modification in SMA through timely gene-targeted intervention contrasts with DMD, where the underlying myofiber pathology remains less amenable to reversal. This comparison highlights both diseases’ evolving therapeutic landscapes while contextualizing our SMA cohort’s clinical trajectory relative to DMD. Integrated, early intervention redefines clinical possibilities in SMA, inducing new phenotypes and substantially altering patient outcomes in a manner not yet replicated in DMD. Multidisciplinary care and precision medicine are pivotal in this therapeutic paradigm shift. These results support the ongoing integration of gene therapy advances for both disorders.

Standards of care include multidisciplinary management that integrates pharmacological treatment with supportive therapies, such as physical rehabilitation, respiratory care, orthopedic interventions and nutritional support [11].

Swallowing dysfunction in spinal muscular atrophy (SMA) reflects the disease’s impact on bulbar and respiratory muscles, posing significant risks such as aspiration pneumonia and malnutrition. The data from this cohort indicate that the vast majority of SMA patients are able to maintain oral feeding, with 89.4% not requiring permanent enteral interventions. This predominance of oral alimentation suggests that, despite the challenges imposed by disease severity—particularly in more affected subtypes—comprehensive multidisciplinary care and advances in swallowing assessment can help preserve functional oral intake for most individuals. The relatively low rates of nasogastric tube (7.7%) and gastrostomy (2.9%) use highlight that invasive nutritional support remains a last-resort measure, reserved for patients with persistent or severe swallowing difficulties. These findings support current SMA management strategies that prioritize the maintenance of oral nutrition whenever feasible, but also underscore the necessity for early, individualized intervention to ensure safe and adequate nourishment, minimize complications, and optimize quality of life.

The analysis of respiratory support requirements and motor function outcomes in SMA patients provides important insights into disease severity and therapeutic response. Non-invasive ventilation and tracheostomy are critical interventions for individuals experiencing respiratory muscle weakness and compromised ventilatory capacity. In this cohort, the need for NIV (35.6%) and tracheostomy (2.9%) reflects the spectrum of respiratory involvement in SMA, with the majority able to remain free of these interventions.

Statistical comparisons indicate a strong link between initial motor impairment and the requirement for NIV. At baseline, patients needing NIV had significantly lower motor scores than those not requiring respiratory support, confirming that severe motor weakness increases the risk of respiratory complications (*p* < 0.01). However, longitudinal follow-up demonstrates that both groups benefited from disease-modifying therapy, with motor scores increasing over time and the between-group difference becoming non-significant (*p* = 0.25). The CHOP INTEND and HFMSE score trajectories reinforce these findings: regardless of respiratory support status, nearly all patients achieved clinically meaningful improvements, and the gap in motor function between NIV and non-NIV patients narrowed substantially by 24 months.

This pattern highlights the transformative impact of modern therapies in SMA, not only in improving motor skills but also potentially in modifying the course of respiratory decline. The more variable but generally positive response in patients requiring NIV underscores the importance of early intervention and ongoing multidisciplinary care. The rarity of tracheostomy further suggests effective prevention of progression to invasive respiratory failure in the current treatment era. Overall, these data support the continued use of motor assessments and prompt respiratory management as key components of SMA care, which together can maximize function and enhance long-term outcomes for affected children.

The longitudinal assessment of motor function in SMA patients undergoing disease-modifying therapies—Nusinersen, Risdiplam, or Onasemnogene Abeparvovec—demonstrates significant, sustained improvements across multiple standardized scales. The progressive increase in CHOP INTEND and HFMSE scores at all timepoints, with each interval showing statistically significant gains, clearly supports the efficacy of these treatments in altering the natural course of SMA. Instead of mere stabilization, most patients achieved continuous, incremental motor improvements, especially in the initial six months of therapy; this trend persisted beyond the first year, indicating that benefits are not limited to early intervention alone.

Importantly, when data are stratified by SMN2 copy number, the analysis reveals that all genotypic subgroups—whether two, three, or four SMN2 copies—exhibited either marked improvements or stabilization in motor milestones following therapy. Higher SMN2 copy numbers remained associated with better outcomes, but the gap between phenotypes narrowed over time as treated patients with historically poorer prognosis also registered substantial gains.

These findings validate the long-term value of disease-modifying therapies, emphasizing both the necessity and effectiveness of early diagnosis and intervention. They also underline the importance of continuous functional assessment to monitor treatment efficacy and optimize patient care. Ultimately, the data point to a new trajectory for phenotypic evolution in SMA: patients are increasingly able to maintain or improve motor abilities over extended follow-up, significantly enhancing function and quality of life compared with the historical, untreated course of the disease.

To enhance the rigor and credibility of characterizing new clinical phenotypes in SMA, future studies should incorporate systematic and objective stratification methods. This might include detailed genotypic profiling, age at therapeutic intervention, longitudinal functional classifications, and structured subgroup analyses based on treatment modalities. Additionally, utilizing advanced biostatistical modeling or clustering approaches could uncover previously unrecognized subtypes within the cohort, lending greater specificity to the “new phenotype” claims. Transparent reporting and analysis of “cross-over” patients—those who switched therapies during the study—as well as thorough documentation and accounting for patients lost to follow-up, are essential steps to minimize bias and support the reproducibility of findings. Prospective, multicenter studies with well-defined endpoints and complete patient tracking would provide a more robust framework for phenotype delineation and reduce the risk of confounding by survivor bias or treatment heterogeneity.

## 5. Limitations

The psychosocial dimensions of patients’ and parents’ experience in SMA are critical in understanding quality of life beyond physical symptoms. These aspects are not included in this cohort and this constitutes a limitation of this research.

The limitations of this research are also phenotypic variability and the influence of unmeasured genetic or environmental modifiers, which remain significant research gaps, as factors beyond SMN2 copy number contribute to clinical heterogeneity in SMA. Future directions for research should include prospective, multicenter studies with standardized, high-quality data collection and longitudinal assessment to better control for bias, account for confounders, and clarify the prognostic impact of genetic and non-genetic modifiers.

## 6. Conclusions

Our findings demonstrate that early and sustained SMN-enhancing therapy leads to marked and durable motor gains in SMA, a clinical trajectory distinct from DMD. This reinforces the importance of early diagnosis and individualized therapy, supported by multidisciplinary care teams. Contextualizing SMA outcomes alongside DMD underscores both successes in gene therapy and persisting challenges in neuromuscular disorders.

Within the current landscape of SMA therapies, it is essential to ensure comprehensive and equitable access to the optimal standards of care to achieve the best outcomes and to describe new phenotypes in patients with SMA. We need a consensus on the SMA type classification and the objectives that determine the effectiveness of any treatment intervention. Standard newborn screening seems to be an appropriate tool to achieve maximum treatment effects, timely diagnosis and initiation of treatment.

This paper provides an overview of how early diagnosis and modern treatments have changed the clinical paradigm of SMA, highlighting the importance of a multidisciplinary approach in the management of this complex disease.

The current landscape of SMA research highlights that early diagnosis coupled with rapidly initiated, targeted pharmacological therapies, backed by comprehensive supportive care and kinetotherapy, leads to notable recovery of motor function and quality of life improvements. Ongoing studies continue to refine these strategies to achieve even better outcomes for all SMA patients.

The development and broad application of disease-modifying therapies have dramatically changed the clinical trajectory of SMA, transforming a previously fatal disorder into a treatable one for many patients. This study points out the real-world therapeutic impact in the European health system and documents and analyzes these changes, fueling ongoing innovations in diagnostics and long-term management strategies. The study cohort are child patients with age and onset diversity and a treatment-experienced SMA population with extended age range and phenotypic variability, reflecting advancements in SMA care and research focus. Romania also has plans and advocacy efforts underway for newborn screening of SMA to enable earlier diagnosis and treatment initiation, which is crucial for improving patient outcomes. Romania is actively developing a structured health system for rare diseases through specialized centers, national programs, and international cooperation, though gaps remain in care coverage and patient integration.

## Figures and Tables

**Table 1:** Cohort characteristic.

Parameter	Overall (*n* = 104 Patients)
Male gender	52 (50%)
Age of onset (months)	48 [9; 106]
SMN2 copies	
2	52 (50%)
3	51 (49%)
4	1 (1%)
Non-invasive ventilation	37 (35.6%)
Gastrostomy	3 (2.9%)
Nasogastric tube	8 (7.7%)
Oral feeding	93 (89.4%)

**Table 2:** Longitudinal changes in motor assessment scores (CHOP INTEND and HFMSE) in treated SMA cohort.

Measure	Time Point	Median [IQR]
CHOP INTEND	Before	23 [16; 32]
CHOP INTEND	6 months	40 [28; 47.8]
CHOP INTEND	12 months	42 [35.2; 52]
CHOP INTEND	18 months	49 [38.5; 56]
CHOP INTEND	24 months	53 [40.7; 56.8]
HFMSE	Before	32 [23.7; 45.7]
HFMSE	6 months	33 [28; 47.3]
HFMSE	12 months	35 [28; 48.7]
HFMSE	18 months	35 [29; 49.3]
HFMSE	24 months	36 [29; 49.3]

**Table 3:** Non-invasive ventilation (NIV) and scores for motor scales (CHOP INTEND+HFMSE) before and after 24 months of disease-modifying therapy.

Parameter	NIV Present (*n* = 37)	NIV Absent (*n* = 67)	*p*-Value
Score before	16 [10; 28]	32 [24; 44]	<0.01
Score 24 months	44 [25; 53]	46 [34; 54]	0.25

## Data Availability

The original contributions presented in this study are included in the article. Further inquiries can be directed to the corresponding authors.
